# An omental mass. Any idea?

**DOI:** 10.1016/j.ijscr.2019.02.019

**Published:** 2019-02-15

**Authors:** G. Mascianà, G.T. Capolupo, F. Carannante, M. Caricato

**Affiliations:** Department of Geriatric Surgery, University Campus Bio-Medico, Via Alvaro del Portillo 21, 00128 Rome, Italy

**Keywords:** Pseudomyxoma, Omental mass

## Abstract

**Introduction:**

Pseudomyxoma extraperitonei (PE) is a rare finding. The most common cause is the rupture of a mucocele of the appendix into the retroperitoneum.

**Presentation of case:**

Here we report a case of a 52 years old female patient with a mass in the right abdomen and vague lower abdominal pain underwent resection of a extraperitoneal encapsulated mass. The histopathological examination revealed a mucinous pseudomyxoma with a low grade of differentiation.

**Discussion:**

We report a case of pseudomyxoma extraperitonei with a review of literature.

**Conclusion:**

The treatment of pseudomyxoma differs substantially depending on whether it is intraperitoneal or extraperitoneal. The risk of recurrence is such that follow-up, based on a physical examination, CT scan and serum markers, is essential.

## Highlights

• Pseudomyxoma peritonei, omental mass, review, abdominal pain, mucinous mass.

## 1 Case presentation

A 52-year-old female patient was referred to our hospital for a mass in the right abdomen and vague lower abdominal pain. The only remarkable event in her past history was a right breast fibroid neoplasm that had been removed 10 years before.

## 2 Investigations

**Fig. 1:**
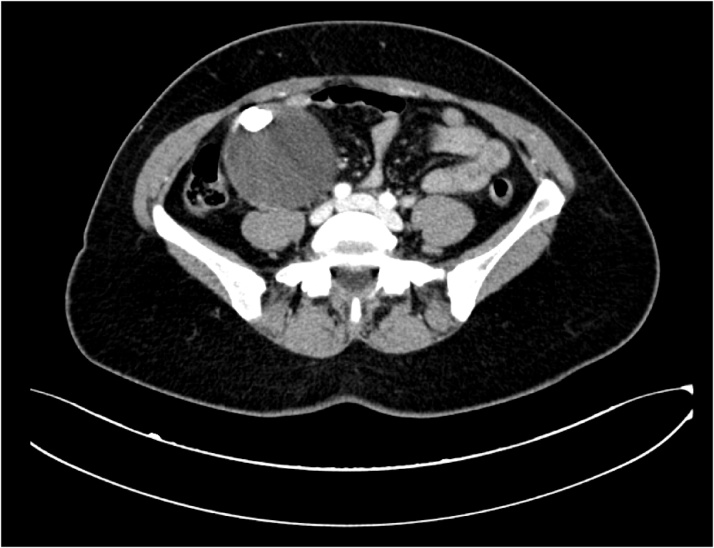
CT scan of the abdomen and pelvis, demonstrating the mass in the right abdomen.

An abdominal examination revealed a large, fixed mass in the right subcostal region. There were no cervical, axillary or inguinal lymphadenopathies, nor any signs of ascites. Other tests were normal with the exception of a slight increase in the CEA level (8.5 ng/ml; normal value: 0–2.5). Computed tomography (CT) of the chest and abdomen revealed a roundish, 100 × 70 × 80-mm mass in the right flank, with regular margins, a partial capsule and a 19-mm internal calcification with mural calcification and hyperdense central striae (Fig. 1). The US-guided aspiration of the mass yielded acellular material. The upper and lower endoscopic examinations were negative.

## 3 Treatment

**Fig. 2:**
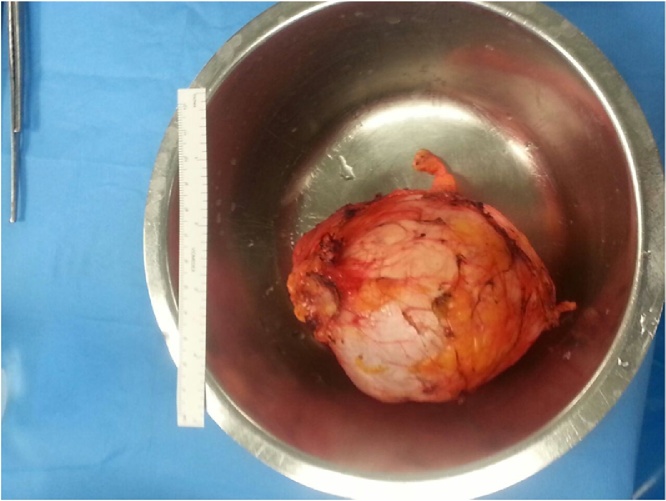
Surgical specimen.

**Fig. 3:**
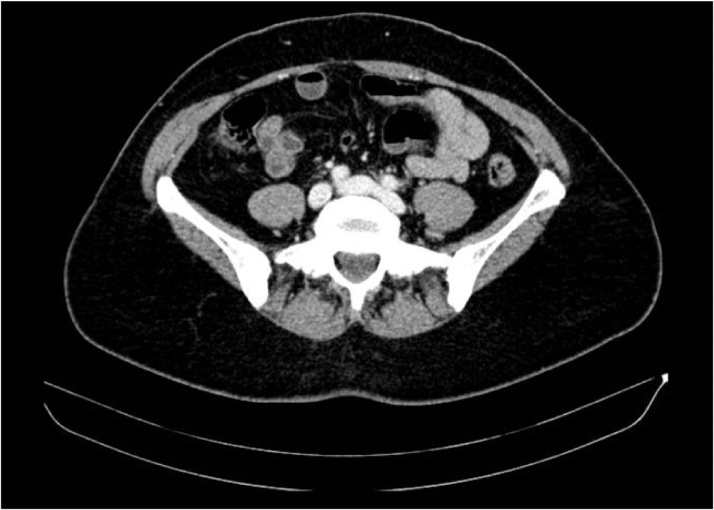
CT scan of the abdomen and pelvis 3 years later, demonstrating no recurrence.

The case was discussed at a multidisciplinary team meeting. As it proved difficult to make a preoperative diagnosis, the patient underwent surgery, during which a peritoneal encapsulated mass that was free of all surrounding structures, including the pancreas, kidneys and bowel, was found. This mass was resected completely without breaking the capsule (Fig. 2). During the surgery, the appendix and ovaries were checked and no correlation with the mass was found. The histopathological analysis revealed a peritoneal mucinous pseudomyxoma with a low grade of differentiation. Immunohistochemistry was positive for CK20 and CK7. The patient was discharged 4 days after surgery without any complications. At follow-up, 3 years later, there was no sign of recurrence (Fig. 3).

## 4 Discussion

Werth [1] first described pseudomyxoma peritonei in 1884 as the presence of mucinous and gelatinous material in the peritoneal cavity. In 1948, Bonann [2] reported a pseudomyxoma involving the retroperitoneum alone, which was subsequently displayed by Coppini [3] in 1950. Twenty years later, Early [4] described a retroperitoneal mucocele of the appendix that contained 10 litres of mucus that had not ruptured, thus allowing a complete curative excision to be performed; this was referred to by Moran [5] as pseudomyxoma extraperitonei (PE) in 1988. Shelton et al. [6] later named it pseudomyxoma retroperitonei. Pseudomyxoma retroperitonei is a rare disease of which there are only 37 reported cases in the literature; the most common cause is the rupture of a mucocele of the appendix into the retroperitoneum (Table 1). It affects both sexes to the same extent, prevalently after the age of 60 years [7], with other potential primary sites including a mucinous neoplasm of the ovary or bowel, or a primary retroperitoneal mucinous cystadenoma/cystadenocarcinoma; a histopathological analysis reveals aggregates of mucus and epithelial cells displaying varying degrees of atypia and differentiation. The cells are generally positive for CK20 and negative for CK7 [8]. Pseudomyxoma can be classified as grade I or benign disseminated peritoneal adenomucinosis, as grade II or intermediate subtype, and as grade III or malignant peritoneal mucinous carcinomatosis [9,10]. In the majority of cases the pathogenesis of pseudomyxoma retroperitonei is explained by a leak through the peritoneum (retroperitoneal presentation associated with intraperitoneal pseudomyxoma). In the absence of peritoneal pseudomyxoma, a variant of the anatomy of the appendix (retroperitoneal location) may explain the extraperitoneal pseudomyxoma, though this hypothesis is still speculative [11]. A preoperative diagnosis is very rare; symptoms such as fatigue, decreased appetite with weight loss, the presence of a palpable mass and slowly progressing abdominal or lumbar pain are common. CEA and CA 19.9 are reported to be increased in 56–75% and 58–67% of patients, respectively. Ultrasound may detect the mucina as retroperitoneal fluid and help to make a diagnosis by means of needle aspiration, while CT with intravenous, oral and rectal contrast may distinguish the mucinous substance from the normal watery fluid by means of density property analysis (5–20 Hounsfield units for mucous vs 0 Hounsfield units for water). At CT, pseudomyxoma retroperitonei appears as a mass that is often multicystic, has septa or thick walls and may be characterized by mural calcifications that displace adjacent structures. [14].

**Table 1:** Case.

Author	Patient (age/sex)	Publication year	Primary tumor	Treatment
Bonnan	37 M	1948	Mucocele of appendix	Evacuation of cyst in 2 stage procedure
Early	57 M	1968	Mucocele of appendix	Debulking of tumour
Early	63 M	1968	Appendix	Excision
Brady	67 M	1986	Appendix	Surgical debulking retroperitoneal chemotherapy
Moran	58 M	1988	NA	Drainage, appendectomy and repeat drainage. Chemotherapy, radiotherapy mucolytic agents, radiofrequency hyperthermia
Baker	33 M	1988	Rectum	Debulking, omentectomy
Snyder	50 F	1992	Appendix	Excision, omentectomy, total hysterectomy and oophorectomy
Fann	47 M	1993	NA	Excision
Radosavljevic	41 M	1993	Appendix	Chemotherapy
Shelton	81 M	1994	Appendiceal mucinous cystadenoma	Debulking and appendectomy
Tamai	39 NA	1995	Appendiceal mucinous adenocarcinoma	Right hemicolectomy, resection of right iliopsoas muscle, partial peritonectomy
Baba	NA	1995	Appendiceal mucinous cystadenoma	NA
Mor	65 M	1996	Appendiceal cystadenocarcinoma	Debulking and appendectomy
Ben-Hur	65 M	1996	Appendix	Excision
Stevens	56 M	1997	Appendiceal mucinous adenocarcinoma	Drainage, radiotherapy
Tsai	69 M	1998	Appendiceal mucinous adenoma	Debulking
Matsuoka	58 F	1999	Mucinous cystadenoma (primary or secondary	NA
Koizumi	46 M	1999	Appendiceal mucinous adenocarcinoma	Right hemicolectomy
Edrees	53 F	1999	Appendiceal mucinous adenocarcinoma	Right hemicolectomy and debulking systemic chemotherapy
Peek	38 M	1999	Appendix	Excision, chemotherapy
Koizumi	53 M	1999	Appendix	NA
Al-Bozom	75 M	2000	Appendix	Chemotherapy
Kojima	78 F	2001	Appendiceal cystadenocarcinoma	Right hemicolectomy
Angelescu	NA M	2001	Mucinous paraenteric cyst	NA
Liu	68 F	2001	Appendiceal mucinous adenocarcinoma	Appendectomy debulking of mucous, intraoperative chemotherapy, systemic chemotherapy
Solkar	57 M	2004	NA	Excision of cyst radiotherapy chemotherapy
Hirokawa	55 F	2004	Ascending colon cancer	Right hemicolectomy oophorectomy, mucous removal. Systematic chemotherapy A
Niwa	80 F	2007	Appendiceal mucinous adenocarcinoma	Resection of mucous and ileum, cecum both ovaries and uterus
Cakmak	51 F	2009	Appendiceal mucinous adenocarcinoma	En block resection with a portion of iliac bone appendectomy, systemic chemotherapy and radiotherapy
Chamisa	48 F	2011	Ovary	Debulking right oophorectomy and chemotherapy
Ioannidis	74 M	2012	Appendiceal mucinos adenocarcinoma	Debulking right hemicolectomy chemotherapy
Ioannidis	68 F	2012	Appendiceal mucinous adenocarcinoma	Debulking right hemicolectomy chemotherapy
Lim	53 F	2014	Appendix	Total abdominal hysterectomy, bilateral salpingo-oophorectomy, omentectomy, appendectomy, and nonoptimal debulking.
Mavrodin	56 F	2014	Appendix	Appendectomy, total hysterectomy with right adnexectomy, omentectomy
Spyropoulos	70 F	2014	Appendix	Right hemicolectomy
	84 M	2014	NA	Inspected and palpated with no recognition of the appendix
Joo	80 F	2015	Ovary	Bilateral salpingooophorectomy with massive adhesiolysis and excision of a mass in the retroperitoneal space.
Martins	66 F	2015	Appendix	Peritoneal lavage, hysterectomy, bilateral salpingo-oophorectomy, omentectomy and appendicectomy

The treatment of pseudomyxoma differs substantially depending on whether it is intraperitoneal or extraperitoneal. For intraperitoneal pseudomyxoma, Sugarbaker et al. [15] recommended an aggressive, complex surgical procedure that involves extirpation of the mucinous material, debulking and peritonectomy in order to remove as much macroscopic disease as possible (cytoreductive surgery, CRS) using heated intraperitoneal hyperthermic chemotherapy (HIPEC). By contrast, the recommended treatment for extraperitoneal pseudomyxoma is, as for benign disease, resection of the site of origin sometimes followed by systematic chemotherapy. Glehen et al. [17] reported a median survival of 156 months, with 5- and 10-year survival rates of 72% and 55%, respectively, in 501 pseudomyxoma peritonei patients who had undergone CRS (complete or incomplete) followed by HIPEC. The majority of the patients (˜70%) underwent complete cytoreduction. This uniform treatment approach has led to a better 10-year survival than that recorded in historical controls [18,19]. Although no data are available on the use of hyperthermic retroperitoneal chemotherapy, this treatment should be considered owing to the high recurrence rate.

The risk of recurrence is such that follow-up, based on a physical examination, CT scan and serum markers, is essential. Combined treatment in pseudomyxoma retroperitonei is associated with a 20-year survival rate in up to 70% of patients [21], whereas the survival rate for pseudomyxoma intraperitonei, in which vital abdominal structures are involved, is shorter [22].

## Conflicts of interest

The authors disclose no conflicts.

## Sources of funding

The Authors disclose no sources of funding for research.

## Ethical Approval

This is a case report and review of literature. It’s exempt from ethical approval.

## Consent

Written informed consent was obtained from the patient for publication of this case report and accompanying images. A copy of the written consent is available for review by the Editor-in-Chief of this journal on request

## Author’s contribution

G.T. Capolupo: study design, data collections, data analysis, and writing.

G. Mascianà: study design, data collections, data analysis, and writing.

F. Carannante: study design, data collections, data analysis, and writing.

M. Caricato: reviewer.

## Registration of research studies

This is not a human study.

## Guarantor

Prof. Marco Caricato.

## Provenance and peer review

Not commissioned, externally peer-reviewed.
